# Baseline status regarding compliance with neo-BFHI recommendations in South African neonatal wards: a cross-sectional survey

**DOI:** 10.1186/s12913-023-09396-6

**Published:** 2023-05-01

**Authors:** Welma Lubbe, Lisa Springer, Ragnhild Maastrup, Laura N. Haiek, Madimetja Nyaloko

**Affiliations:** 1 NuMiQ Research Focus Area, North-West University South Africa, Potchefstroom, South Africa; 2 Department of Neonatology, Copenhagen University Hospital Rigshospitalet, Copenhagen, Denmark; 3 Ministère de la Santé et des Services sociaux, Direction générale de la santé publique, Quebec, Canada; 4 Department of Family Medicine, McGill University, Montreal, Quebec, Canada; 5 St. Mary’s Hospital, St. Mary’s Research Centre, Montreal, Quebec, Canada

**Keywords:** Baby-Friendly Hospital Initiative, Breastfeeding, Compliance, Neonatal wards, Infant, Kangaroo mother care

## Abstract

**Background:**

In 2009, the World Health Organization and the United Nations Children’s Fund issued a revised Baby-friendly Hospital Initiative (BFHI) package to encourage all healthcare facilities to promote the advice of exclusive breastfeeding. The scope of the BFHI was expanded to include neonatal units by the Nordic and Quebec Working Group.

**Aim:**

To determine the level of compliance with the recommendations outlined in the “Baby-friendly Hospital Initiative for neonatal wards” (Neo-BFHI) in the South African neonatal wards.

**Method:**

In this cross-sectional survey, the sample included neonatal wards (N = 33) from public and private hospital facilities. Using EasyTrial software, the Neo-BFHI self-assessment questionnaire was utilized to collect the data. The data was transferred to MS Excel (version 15.0.5127.1000) and analysed with the Statistical Package for Social Sciences version 24.

**Results:**

The South African median score for Neo-BFHI compliance was 77. Neonatal wards in public hospitals scored higher (85) than those in private hospitals (73). Neonatal wards in hospitals that were accredited Baby-friendly had higher compliance scores than those without accreditation. The country had the highest compliance scores (100, 90) on Guiding Principle 1 (respect towards mothers) and step 5 (breastfeeding support), respectively. However, it scored low (71, 58) on steps 4 (enhancing kangaroo mother care) and 7 (maternal infant “togetherness”), respectively. Level 1 and 2 care facilities scored significantly higher than level 3.

**Conclusion:**

Although South Africa successfully implemented the Neo-BFHI recommendations, private hospitals had a smaller number of BFHI-accredited facilities and lower compliance than public hospitals. Strategies should be developed to strengthen and improve BFHI accreditation and compliance, particularly in private hospitals.

## Introduction and background

Exclusive breastfeeding (EBF) is an optimal way of feeding infants aged six months or less worldwide. The World Health Organization (WHO) defined EBF as providing breast milk to an infant as their sole source of nutrition directly or indirectly from the breast while also allowing for the administration of prescribed medications, vitamins, and minerals [1]. It is evident that EBF holds health benefits for infants, mothers, and community members [2]. For instance, while breast milk provides ideal nutrients and protective antibodies for the infant, which consequently yields short-, medium-, and long-term health outcomes for babies, breastfeeding reduces the incidence of developing ovarian and breast cancer for mothers [3, 4].

Due to the benefits of exclusive breastfeeding, the Baby Friendly Hospital Initiative (BFHI) campaign was initiated worldwide in 1991 to promote breastfeeding. The BFHI was originally designed by WHO and UNICEF in 2009 and updated several times [5, 6] to ensure the breastfeeding success of healthy newborn infants born from healthy mothers and recommended policies and practices in delivery and maternal wards. By January 2017, the BFHI program was adopted as a national policy by 117 countries, including South Africa [7]. However, the benefit of expanding the BFHI to include neonatal wards became evident, and in 2015, a Nordic and Quebec Working Group launched the Baby-Friendly Hospital Initiative for neonatal wards (Neo-BFHI). In 2020, the WHO published a document with similar recommendations to protect, promote and support breastfeeding of small, sick, and preterm newborns [6].

The proposed Neo-BFHI included Three Guiding Principles and Ten Steps to Successful Breastfeeding (**See** Table 1) to protect, promote, and support breastfeeding and adherence to the International Code of Marketing of Breast-milk Substitutes [8]. A global survey with 36 countries was conducted using the Neo-BFHI Self-Assessment questionnaire allow assessment of policies and practices supporting breastfeeding in neonatal wards [9]. South Africa participated in the international study and therefore used the same methodology, making it possible for South Africa to situate their results from a global perspective.

**Table 1:** The components of the Neo-BFHI

**Three guiding principles**	
GP 1	Staff attitudes toward the mother must focus on the individual mother and her situation
GP 2	The facility must provide familycentered care supported by the environment.
GP 3	The health care system must ensure continuity of care from pregnancy to after the infant’s discharge.
**Expanded Ten Steps to Successful Breastfeeding**	
Step 1	Have a written breastfeeding policy that is routinely communicated to all healthcare staff.
Step 2	Educate and train all staff in the specific knowledge and skills necessary to implement this policy.
Step 3	Inform hospitalized pregnant women at risk for preterm delivery or birth of a sick infant about the benefits of breastfeeding and the management of lactation and breastfeeding.
Step 4	Encourage early, continuous, and prolonged mother-infant skin-to-skin contact/ Kangaroo Mother Care.
Step 5	Show mothers how to initiate and maintain lactation and establish early breastfeeding with infant stability as the only criterion.
Step 6	Give newborn infants no food or drink other than breast milk unless medically indicated.
Step 7	Enable mothers and infants to remain together 24 h a day.
Step 8	Encourage demand breastfeeding or, when needed, semi-demand feeding as a transitional strategy for preterm and sick infants.
Step 9	Use alternatives to bottle feeding, at least until breastfeeding is well established, and use pacifiers and nipple shields only for justifiable reasons.
Step 10	Prepare parents for continued breastfeeding and ensure access to support services/groups after hospital discharge.
**Code**	
	Compliance with the International Code of Marketing of Breast-milk Substitutes and relevant World Health Assembly resolutions

*GP*: Guiding principle

South Africa had an EBF prevalence at six months of 32% in 2016 [10], which is lower than the global rate of 38% [11]. Scaling up the EBF rate to a prevalence target of at least 50% as proposed by WHO [12], the Neo-BFHI recommendations, policies, and practices warrant assessment to ensure initiation, continuation, and maintenance of EBF not only in neonatal wards but also post-discharge from hospital facilities. Furthermore, South Africa could benefit from participating in the international study since about 14% of South African infants are born with low birth weight and prematurity and admitted to neonatal wards [13]. In contrast, little is known regarding EBF compliance in these wards. This study aimed to determine the compliance level of Neo-BFHI recommendations in South African neonatal wards and compare the Neo-BFHI compliance between different levels of neonatal care, including public and private, BFHI accredited and non-accredited hospital facilities.

## Methods

A cross-sectional study of breastfeeding policies and practices was conducted in South African neonatal wards. South Africa is a Southern African country with a population of approximately 60 million people [14]. It is classified as a World Bank Group 3 country [15], comprising public and private healthcare establishments. In the healthcare system, there are three levels of care: low intensive care (level 1), intermediate care (level 2), and high intensive care (level 3) [16]. All private hospitals are accredited to provide care on a level 3 basis.

### Population

The study population was drawn from South African neonatal wards. At the time of the study, there was no documented and publicly available information detailing the number, types, and levels of neonatal wards in public and private hospital facilities in South Africa. Based on previous studies [17–19], 356 neonatal wards were identified.

### Inclusion criteria

All neonatal wards in South African public and private hospitals, regardless of their BFHI accreditation status, were eligible for inclusion. For this study, neonatal wards included general neonatal wards, high-care neonatal units, kangaroo mother-care units, and neonatal intensive care units.

### Sample size

The sample included all South African neonatal wards. Out of the 356 neonatal wards, public and private hospital authorities consented to 126 neonatal wards participating in the survey. However, fifty-seven (n = 57) neonatal wards (representatives) did not grant goodwill permission, yielding a sample size of sixty-nine (n = 69) who provided the researchers with the manager’s e-mail address.

Data Collection Instruments.

The Neo-BFHI Self-Assessment questionnaire was used to determine South Africa’s compliance with Neo-BFHI policies and practices. The questionnaire was adopted from the Neo-BFHI Self-Appraisal tool [8], which was modelled after the WHO/UNICEF BFHI package, “Sect. 4: Hospital Self-Appraisal and Monitoring” [1]. The original versions of the questionnaire were developed in both English and French. Existing questions were converted into statements, and most yes/no answer choices were replaced by a 5-point Likert scale [9]. In total, 63 indicators were provided, ranging from two to 10 for each of the three Guiding Principles, Ten Steps, and Code [9]. The content and face validity were affirmed in four countries by 11 experts by confirming the relevancy and accuracy of the tool [9]. No alterations were made to the original version.

### Data collection procedure

The Neo-BFHI Self-Assessment questionnaire was imported into EasyTrial Online survey software for data collection purposes. This software consists of clinical trial tasks (operational and logistical) designed to maximise the efficiency of healthcare professionals [20]. All the names of the private and public hospital facilities (n = 126) that consented to participate in the survey were registered in the EasyTrial software for data management. However, the URL link of the Neo-BFHI self-assessment questionnaire was distributed on August 31^st,^ 2017, through secured e-mail to only neonatal ward representatives (n = 69) that granted the goodwill permission. The managers were asked to ensure that the questionnaire was completed together with the healthcare professional(s) in their ward with the most knowledge of breastfeeding practices. Each ward should answer one questionnaire. Approximately one hour was required to complete the questionnaire. Respondents were, however, able to exit and return at any moment to continue or complete the survey. The online questionnaire required the completion of all fields to be submitted. The deadline given to respondents to submit the online questionnaires was December 31st, 2017.

The international study leader (RM) had EasyTrial online access to track all questionnaires distributed, completed, uncompleted, and returned. Following the initial e-mail message, reminders to complete and return the questionnaires were sent out on weeks three, five, and every six weeks. Confidentiality was ensured by allocating each neonatal ward with a unique identification code, kept within the EasyTrial database, and only used to prepare the personalized benchmark reports. EasyTrial online software automatically deactivated the URL link to stop the submission of questionnaires on the 31st of December 2017.

### Data analysis

After the deadline for submission of the online questionnaires, the international study leader exported the raw data from EasyTrial to MS Excel (version 15.0.5127.1000) with unique codes. The exported data was sent to the South Africa team via secured e-mail for further analysis. The data analysis was conducted at the international study level following the identical methodology provided by Maastrup et al. [9]. The five-point Likert scale responses (None to All or Never to Always) corresponded to a numerical value of 0-25-50-75-100 points. “Yes” was equivalent to 100 points in the Yes/No responses, while “no” and “don’t know” were equal to zero points. In total, nine of the 63 indicators were quantified using multiple statements. In these instances, the indicator’s points were calculated as the mean of the values for each statement. Three other indicators were given grades based on how well they met the requirements. They were considered to meet the criteria when they met the minimum level.

Each neonatal ward’s compliance was determined as the mean value obtained from each indicator evaluating the three Guiding Principles, the Ten Steps, and the Code, resulting in 14 ward partial scores. The overall score was then calculated using the mean of the ward’s partial scores. Country partial scores were computed as the medians of the 14 ward partial scores, while country overall scores were derived as the respective ward overall scores. When an indicator was unable to quantify a practice, it was deemed inapplicable and so did not add value to the score. All scores were assigned a value between 0 and 100 [9]. Higher scores indicated better compliance, and ideal scores were 100.

Data were also analysed using descriptive and inferential statistics presented as mean and median values. Country, level of care/facilities, and private and public hospital facilities scores were calculated as median scores using all neonatal ‘wards’ scores. The Chi-square test was used to determine the comparison across the distribution of categorical variables. Statistical significance was defined as p-values less than 0.05 [21].

## Presentation of findings

### Characteristics of respondents

Twenty-six neonatal wards (n = 26) from private and seven public hospitals (n = 7) participated in the study. Public hospitals were represented by one level 1, two level 2, and four level 3 facilities. Private hospitals were represented by 26 level 3 facilities (classified as level 3 in South Africa). Out of 69 neonatal ‘wards’ representatives, only 33 returned the completed questionnaires, resulting in a response rate of 48% (**see** Table 2).

**Table 2:** Characteristics of respondents

	Private hospitals	Public hospitals	Overall hospitals
Number of hospitals with neonatal wards	104	252	356
Level 1 facilities	-	188	188
Level 2 facilities	-	42	42
Level 3 facilities	104	22	126
The number of hospitals contacted	40	93	133
Number of hospitals that granted goodwill permission	40	29	69
Number of neonatal wards that received questionnaires (one per hospital)	40	29	69
Number of neonatal wards responded (one per hospital)	26	7	33

### Compliance scores

The overall country score for neonatal wards in South Africa was 77, with level 2 facilities scoring the highest median score (92) and level 3 facilities achieving the lowest median score (75). Public hospitals’ overall score was higher (85) as compared to the private hospital group score (74), (**see** Table 3).

**Table 3:** Partial median scores and overall South African median score (facility levels, private and public hospital facilities)

		GP 1	GP 2	GP 3	S1	S2	S3	S4	S5	S5	S7	S8	S9	S 10	C	OS
Number of indicators (Total = 63)		2	6	3	4	5	2	6	10	2	3	4	5	4	7	
Level 1	(*N* = 1)	100	82	100	100	98	100	94	100	75	83	75	40	100	92	89
Level 2	(*N* = 2)	88	85	96	83	93	94	92	99	100	88	97	93	88	93	92
Level 3	(*N* = 30)	100	79	92	67	81	75	67	88	88	50	81	83	75	82	75
South African score	(*N* = 33)	100	82	92	75	84	81	71	90	88	58	81	85	75	82	77
Public hospitals	(*N* = 7)	86	76	88	81	92	75	86	90	95	82	84	80	83	94	85
Private hospitals	(*N* = 26)	94	80	86	46	74	65	65	86	88	50	82	81	70	74	74

*Note*. *GP*: Guiding Principles; *S*: Step; *C*: Code; *OS*: Overall score; *N*: Number of wards

27% (public hospitals N = 5; private hospitals N = 4) of the wards were in BFHI-accredited hospitals with an overall mean compliance score of 91, whereas 30% of the respondents (public hospitals N = 2; private hospitals N = 8) indicated the intention of attaining BFHI accreditation for their neonatal wards. The overall compliance score of non-accredited hospitals was 73. In BFHI-accredited public and private hospitals, the compliance rate was higher (91) than in non-accredited public (73) and private hospitals (72) (**See** Table 4). This is a strong observation supporting the need to enforce Neo-BFHI accreditation to all neonatal units.

**Table 4:** Partial median compliance score of neonatal wards of accredited and non-accredited BFHI hospitals

	Public hospitals		Private hospitals		Overall hospitals	
	Accredited BFHI (N = 5)	Non-accredited BFHI (N = 2)	Accredited BFHI (N = 4)	Non-accredited BFHI (N = 22)	Accredited BFHI (N = 8)	Non-accredited BFHI (N = 25)
GP/Step/Code						
GP1	95	63	100	93	98	78
GP2	80	64	95	78	88	71
GP3	95	71	96	86	96	79
Step 1	100	67	94	39	97	53
Step 2	96	83	95	70	96	77
Step 3	85	50	91	60	88	55
Step 4	91	76	80	63	86	70
Step 5	95	79	91	85	93	82
Step 6	95	94	94	87	95	91
Step 7	88	67	73	46	81	57
Step 8	88	75	91	80	90	78
Step 9	77	87	85	80	81	84
Step 10	89	69	86	67	88	68
Code	100	82	97	70	99	76
Overall mean	91	73	91	72	91	73

*Note*. BFHI: Baby-friendly Hospital Initiative; *GP*: guiding principle; *N*: number of hospitals

Overall, Guiding Principle 1 had the highest compliance score (100), followed by Guiding Principle 3 (92) and Guiding Principle 2 with the lowest score (82). The highest score for Guiding Principles in public hospitals was Guiding Principle 3 (88), of which private hospitals scored slightly lower (86), indicating that continuity in care from pregnancy to post-discharge rendered in both public and private hospitals were more or less the same. On the other hand, the private hospital facilities’ staff demonstrated respect towards mothers with a score of (94) compared to that of public hospital facilities with a lower score (85) (**see** Table 3). There was no significant difference among compliance scores of Guiding Principle 1 [*X*^*2*^(4, N = 33) = 2.09, p = .72] and principle 3 [*X*^*2*^(4, N = 33) = 6.99, p = .23] in public and private hospitals.

Among the 10 Neo-BFHI Steps, Step 5, regarding breastfeeding establishment and maintenance, had the highest country partial score (90), followed by step 6, about exclusive breastfeeding (88). The level 1 facility scored 100 in step 5, and level 2 facilities scored 100 in step 6 (**See** Table 3). Statistically, there was a significant difference in step 5 and step 6 compliance scores between the public and private hospitals [*X*^*2*^(2, N = 33) = 12.56, p = .02. Furthermore, there was a significant relationship between BFHI-accredited hospitals and step 6 [*X*^*2*^(3, N = 33) = 8.88, p = .03]. At the same time, none was found in step 5 [*X*^*2*^(14, N = 33) = 17.79, p = .22].

The two steps with the lowest country partial scores were Steps 7 and 4 (58, 71). Level 3 facilities had the lowest scores in both Step 7 about mother and infant “togetherness” (50) and step 4, which dealt with Kangaroo Mother Care (67). In Step 4, the public hospital level 3 facilities scored 88, while private hospital groups scored 65. The scores did not differ significantly by hospital type, *X*^*2*^(3, N = 33) = 4.96, p = .18. Furthermore, in Step 7 again, public hospital level 3 facilities scored higher (82) than level 3 in private hospitals (50). However, unlike in Step 4, there was a significant difference between the scores in Step 7 in both public and private hospitals [*X*^*2*^(3, N = 33) = 9.86, p = .02]. BFHI accreditation status did not influence step 4 or 7 scores, p > .05.

The Code had a country-partial score of 82. The level 1 and 2 facilities had the highest code scores of 92 and 93, respectively, compared to level 3 facilities, which had the lowest score of 82. Public hospital facilities scored higher (94) than private hospital facilities (74) (**see** Table 3).

## Discussion

This study aimed to establish the compliance level of Neo-BFHI recommendations in South African neonatal wards and compare the Neo-BFHI compliance between different levels of care, including private and public, BFHI accredited and non-accredited hospital facilities. The median compliance score of South African hospitals indicated a rather high level of Neo-BFHI compliance. Public hospital facilities had greater Neo-BFHI compliance than private hospital facilities. Care facilities at levels 1 and 2 scored higher than those at levels 3.

The study found high levels of adherence to Guiding Principle 1 regarding treating mothers with respect with little variations across the level of care facilities. This finding might be due to the ‘Patients’ Rights Charter, which promotes respect among patients and healthcare workers. Consistent with this finding, previous studies conducted in Oromia [22] and Ethiopia [23] showed that mothers were satisfied with the respect and courtesy given to healthcare staff.

The country’s score (77) was equal to the overall international level score, published in the first international survey reporting Neo-BFHI compliance rates in 917 neonatal wards across 36 countries [9]. In South Africa, not all neonatal wards of participating hospitals were BFHI-accredited. Nevertheless, the country’s score (77) indicated that participating hospitals applied the Neo-BFHI recommendations to a certain extent. The basis for compliance in the absence of a BFHI policy could be the role of “The Tshwane Declaration of support for breastfeeding in South Africa” [24]. The Tshwane Declaration’s role includes supporting and enhancing measures to ensure the promotion of breastfeeding. This may also account for the high compliance with step 5 concerning the commencement and maintenance of breastfeeding. Consequently, this data suggests that the Tshwane Declaration may have supported implementing BFHI policies and practices in South African neonatal wards.

The BFHI program can be adapted to various cultural contexts [25]. The WHO specified in the BFHI’s core messages that BFHI should address public and private facilities [26]. The current study supported WHO by including private and public hospitals in the final sample. The findings indicate that public hospital facilities consistently scored higher than private hospitals across all Guiding Principles, Ten Steps, and Codes. This is unexpected given the common perception that private hospitals are well-equipped and resourced compared to overburdened public facilities. This variation might be because more public hospitals hold BFHI accreditation than private ones, allowing them to provide an advanced level of BFHI services. Furthermore, the higher scores of levels 1 and 2 (which are only in public hospitals) could have contributed to the higher scores in public hospitals. To ensure the coverage application of policies and practices to protect, promote, and support breastfeeding in all neonatal wards, it is suggested to expand and expedite BFHI accreditation to private hospital institutions.

The current study revealed that level 1 and 2 care facilities had higher compliance scores of 89 and 92, respectively than level 3 care facilities. Because Level 1 and 2 facilities were situated in public hospital facilities, this implies that the public facilities performed higher than private facilities in initiating and maintaining breastfeeding and ensuring exclusive breastfeeding. This compliance variation across level 3 care facilities is expected owing to the scope of all the facilities. The critically sick neonates are transferred to level 3 facilities which provide more specialised care. The referral system, disrupting the continuity of care, might have contributed to this low score for level 3 hospitals. These findings add to those of MacPhee et al. [27], indicating that enormous workloads negatively impact the quality of care provided by nurses. This may explain the low compliance with components of the Neo-BFHI Guiding Principles and Steps in level 3 care facilities.

## Strengths and limitations

The strength of the study was the participation of neonatal wards from both public and private hospitals.

Due to the low response rate, an accurate representation of the Neo-BFHI compliance rate in the country could not be achieved. Obtaining goodwill permission from public hospital facilities required consultation with multiple authorities, who each had their own rules and processes, negatively influencing recruitment. Also, the fact that the survey relied on a self-assessment questionnaire completed by healthcare professionals posed a risk of bias which might have impacted the true reflection of compliance with the Neo-BFHI. These limitations may have a limiting impact on the generalization of the findings. Another limitation was the small number of level 1 and 2 wards and neonatal wards in BFHI-accredited hospitals. The lack of significant differences between wards in private and public BFHI-accredited hospitals could be due to a lack of power. Still, the findings point in the same direction as the international study [9].

## Conclusions

Altogether, South Africa effectively implemented the Neo-BFHI recommendations, achieving a compliance rate of 77. This overall score included both public and private hospital facilities. The private hospitals had a smaller number of BFHI-accredited facilities and lower compliance than public hospitals. However, the findings indicate that neonatal wards in South Africa, including ones in private facilities, support breastfeeding and implement the Neo-BFHI recommendations to a certain extent, even without a policy specifically targeting the neonatal wards. It is strongly recommended that all non-accredited facilities seek BFHI accreditation. Furthermore, strategies for strengthening and improving compliance in all BFHI-accredited neonatal wards throughout South Africa should be developed.

## Data Availability

The dataset used and analysed in this manuscript is available from the corresponding author upon reasonable request.

## Declarations

### Disclaimer

The views expressed in this manuscript are of the authors, therefore, do not reflect those of any official organisation.

## List of Abbreviations

BFHI: Baby-friendly Hospital Initiatives
Neo-BFHI: Baby-friendly Hospital Initiatives for neonatal wards
WHO: World Health Organization
EBF: Exclusive breastfeeding
UNICEF: United Nations ‘Children’s Fund
